# The knowledge and perceptions of healthcare workers regarding obstetrical anal sphincter injuries. A practice audit from a resource-constrained setting

**DOI:** 10.1016/j.xagr.2021.100021

**Published:** 2021-09-25

**Authors:** Randall Kegan Hammond, Thinagrin Dhasarathun Naidoo

**Affiliations:** 1 Department of Obstetrics and Gynaecology, Greys Hospital, Pietermaritzburg, South Africa; 2 Nelson R Mandela School of Medicine, University of KwaZulu-Natal, Durban, South Africa

**Keywords:** knowledge, obstetrical anal sphincter injuries, perineal, resource-limited, training, trauma

## Abstract

**BACKGROUND:**

We hypothesized that knowledge regarding obstetric anal sphincter injuries amongst healthcare workers in our setting is limited. A lack of knowledge would result in worsened clinical outcomes and proving this knowledge deficiency would allow us to institute educational programs to improve outcomes.

**OBJECTIVE:**

This study aimed to assess the knowledge and perceptions of healthcare workers regarding obstetrical anal sphincter injuries in a resource-limited setting.

**STUDY DESIGN:**

Questionnaires assessing the knowledge in classification, diagnosis, and management of obstetrical anal sphincter injuries were completed by 290 doctors and nurses involved in conducting vaginal deliveries at various levels of care (primary, district, regional, tertiary) in a resource-limited setting. Moreover, confidence in managing obstetrical anal sphincter injuries was assessed.

**RESULTS:**

Although the healthcare workers’ knowledge of anatomy was poor, most healthcare workers knew how to define obstetrical anal sphincter injuries and classify these injuries. Most healthcare workers considered obstetrical anal sphincter injuries serious complications and perceived that patients with obstetrical anal sphincter injuries were best managed at a regional- or tertiary-level hospital. There was variation in choice of suture material and methods of repair, with most healthcare workers lacking confidence in managing obstetrical anal sphincter injuries and 96.9% of healthcare workers indicating a need for further training. Most healthcare workers felt that perineal support was the best intrapartum preventative strategy against obstetrical anal sphincter injuries.

**CONCLUSION:**

Here, it was likely that knowledge and confidence in managing obstetrical anal sphincter injuries in most resource-limited settings were suboptimal, highlighting a need for ongoing training.

**AJOG Global Reports at a Glance**

**Why was this study conducted?**

Previous global studies have demonstrated deficits in knowledge of healthcare workers (HCWs) regarding obstetrical anal sphincter injuries (OASIS). No study about OASIS has been conducted in the South African setting.

**Key findings**

The study highlighted that in resource-limited settings, HCWs’ knowledge regarding the diagnosis and management of OASIS is limited.

**What does this add to what is known?**

The study further highlighted the need for ongoing training regarding the diagnosis and management of OASIS and the potential to improve clinical outcomes, particularly in resource-limited settings.

## Introduction

Obstetrical anal sphincter injuries (OASIS) after vaginal birth are estimated to complicate up to 11% of vaginal deliveries worldwide.^1^ A study conducted by Naidoo et al^2^ in 2015 found the incidence in Durban, South Africa, to be 4.1%.

These injuries involving the anal sphincter complex, comprising third- and fourth-degree perineal tears sustained during labor, have been classified by Sultan et al^3^ as follows:

1. 3a involves <50% of the external anal sphincter.
2. 3b involves >50% of the external anal sphincter.
3. 3c involves the internal anal sphincter.
4. Fourth-degree tears involve all of the above and extend to the anal mucosa.

Complications from such injuries result in both physical and psychological morbidities, including pain, urinary and anal incontinence, pelvic organ prolapse, sexual dysfunction, and feelings of depression or anxiety.^2^

Evidence suggests a deficiency in the understanding of perineal anatomy by midwives and trainee doctors.^4^ In a study examining the knowledge regarding sphincter injuries in the United Kingdom by Fernando et al^5^ in 2002, the authors found that 33% of obstetrical consultants and 22% of trainees incorrectly classified the degree of injury. Significant variation of preferred techniques for repair was shown, with 47.8% of consultants preferring end-to-end repair compared with 50.1% of consultants preferring the overlap technique (*P*<.001). Moreover, the study noted that 64% of consultants and trainees felt that their training in the management of OASIS was unsatisfactory. In an Australian study examining perceptions of doctors and midwives regarding their practice and management of the perineum in preventing perineal trauma by East et al,^6^ the authors found that only 77% of doctors reported that they were confident in the diagnosis and management of OASIS, despite having been trained in diagnosing OASIS. Only 13% of midwives felt very confident despite 71% of them having been trained to make the diagnosis. Furthermore, all doctors who participated in the study reported having been trained in perineal repair, but only 88% of doctors were very confident performing the procedure, highlighting that training does not always translate into confidence. However, Zimmo et al^7^ in their study in Palestine in 2017 demonstrated significant improvement in the knowledge after expert training, emphasizing the value of quality training.

The studies highlighted above demonstrated a definite gap in the knowledge and training of healthcare workers (HCWs) regarding the appropriate diagnosis and management of OASIS. However, most studies were performed in well-resourced countries, and little of this subject is known in resource-limited settings, such as South Africa. This study was designed to address the hypothesis that knowledge and perceptions of HCWs on OASIS in a resource-limited setting are limited.

## Materials and Methods

An observational cross-sectional descriptive study was conducted. Doctors and midwives involved in the delivery and obstetrical care of patients were invited to participate.

In an HCW population of 1000, if we assumed that correct knowledge is 50% at a 95% confidence interval with 80% power and a design effect of 1 for random sampling, the estimated minimum sample size calculated was 278.

Doctors included interns, medical officers, registrars, and specialists. The study was conducted at 11 government healthcare facilities in the province of KwaZulu-Natal, South Africa. These include 3 tertiary centers (Greys Hospital, Inkosi Albert Luthuli Central Hospital, and King Edward VIII Hospital), 3 regional hospitals (Edendale Hospital, Addington Hospital, and RK Khan Hospital), 3 district hospitals (Northdale Hospital, Church of Scotland Hospital, and Wentworth Hospital), and 2 community health centers (Imbalenhle Community Health Centre and Richmond Clinic). Furthermore, private specialists working at various private hospitals in KwaZulu-Natal participated and made up 5.2% of the study participants.

Purposive sampling was used in conducting the study. Participants were invited to complete a self-administered questionnaire in English. Data about the HCWs’ knowledge regarding perineal anatomy and the classification and management of OASIS and the level of confidence in managing these injuries were collected. A pilot study was initially conducted, which demonstrated that participants understood all questions.

A descriptive statistical analysis of the data was done. Frequencies and means with standard deviations were presented for categorical and continuous data. A *P* value of <.05 was considered to represent statistical significance.

Regulatory, provincial, and institutional approvals were obtained from the relevant authorities before commencing data collection. Ethical approval was granted by the Biomedical Research Ethics Committee of the University of KwaZulu-Natal (reference number BE 040/18). Before participation in the study, participants signed an informed consent form, and confidentiality was maintained at all times. The principal investigator was responsible for capturing all data.

## Results

Here, 290 questionnaires were completed by HCWs who met the inclusion criteria. The nursing staff made up 37.2% of the respondents. Moreover, interns accounted for 29.3% of the doctors that participated, whereas specialists accounted for 13.1% of participants. Specialist trainees made up 7.2% of study participants. Of the respondents, 34.8% worked in a regional hospital, with 44.5% having <3 years of obstetrical experience (Table 1).

**Table 1:** Respondent designation, place of work, and obstetrical experience

Respondent details	n (%)
Professional designation	
Registered midwife	47 (16.2)
Advanced midwife	41 (14.1)
Professional nurse	20 (6.9)
Intern first year	36 (12.4)
Intern second year	49 (16.9)
Medical officer with >5 y of experience	22 (7.6)
Medical officer with <5 y of experience	16 (5.5)
Registrar first and second year	12 (4.1)
Registrar third and fourth year	9 (3.1)
Specialist	38 (13.1)
Facility type	
CHC and MOU	39 (13.4)
District hospital	50 (17.2)
Regional hospital	101 (34.8)
Tertiary hospital	86 (29.7)
Private hospital	15 (5.2)
Years of experience (y)	
0–3	129 (44.5)
3–5	30 (10.3)
5–10	70 (24.1)
>10	60 (20.7)

*CHC*, community health hospital; *MOU*, memorandum of understanding.

Only 31.4% of HCWs correctly acknowledged that the anal sphincter is made up of 2 muscle layers. However, 69% of HCWs could correctly identify the perineal body on an anatomic illustration. Although 64.5% of respondents could correctly classify third-degree tears, only 44.5% knew the correct subclassification of these injuries. Moreover, 71% of respondents correctly defined OASIS as being a perineal tear, which causes complete or partial disruption of the anal sphincter complex. However, 11.7% of respondents incorrectly stated that complete disruption of the sphincter complex was necessary, whereas 14.8% of respondents reported that there had to be involvement of the anal mucosa (Table 2).

**Table 2:** Knowledge regarding definition, anatomy, and classification

Query	n (%)
What is the definition of an obstetrical anal sphincter injury?	
a. A perineal tear causing complete disruption of the anal sphincter complex	34 (11.7)
b. A perineal tear causing partial or complete disruption of the anal sphincter complex	206 (71.0)
c. A perineal tear that extends involving the anal mucosa	43 (14.8)
d. None of the above	5 (1.7)
The anal sphincter is made up of how many muscle layers?	
a. 1	9 (3.1)
b. 2	91 (31.4)
c. 3	140 (48.3)
d. 4	48 (16.6)
Correct identification of perineal body in anatomic illustration:	
a. Internal anal sphincter	23 (7.9)
b. Perineal body	200 (69.0)
c. Perineal membrane	35 (12.1)
d. Bulbospongiosus muscle	28 (9.7)
A third-degree tear involves which of the following structures?	
a. The perineal skin and perineal muscles	9 (3.1)
b. The perineal skin, perineal muscles, and the anal sphincter	187 (64.5)
c. The perineal skin, perineal muscles, anal sphincter, and anal mucosa	88 (30.3)
d. The perineal skin and vaginal mucosa	4 (1.4)
Third-degree tears may be subclassified as:	
a. 3a and 3b	69 (23.8)
b. 3a, 3b, and 3c	129 (44.5)
c. 3a, 3b, 3c, and 3d	38 (13.1)
d. No subclassification for third-degree tears exists	48 (16.6)
Fourth-degree tears involve the anal mucosa?	
a. True	256 (88.3)
b. False	32 (11.0)

Most HCWs (80.7%) recognized OASIS as a serious complication, with the potential to cause short, intermediate, and long-term morbidities, 14.8% of HCWs reported that the injuries had the potential to cause morbidity to some degree, and the remaining participants were either uncertain or thought they brought no potential harm.

Concerning reducing the occurrence of OASIS, 61.0% of participants thought that supporting the perineum during delivery of the fetal head was the most effective, whereas 30.0% of participants proposed that episiotomy was the most effective.

Digital anorectal examination was correctly identified by 76.2% of HCWs as the most appropriate choice of initial assessment of the degree of injury, and 54.1% of HCWs stated that the most appropriate setting for OASIS repair was either a regional or tertiary hospital with 84.5% correctly noting that the repair should be performed in surgical theater by a skilled individual (Table 3).

**Table 3:** Interventions to reduce obstetrical anal sphincter injuries, appropriate initial assessment, and management

Interventions	n (%)
Should OASIS be considered a serious complication of vaginal delivery?	
a. No. They have low potential to cause short, intermediate, and long-term morbidities.	5 (1.7)
b. To some degree. They have some potential to cause short, intermediate, and long-term morbidities.	43 (14.8)
c. Yes. They have high potential to cause short, intermediate, and long-term morbidities.	234 (80.7)
d. Uncertain. I am not sure of the significance of an OASIS.	6 (2.1)
Which of the following interventions best help reduce anal sphincter injuries?	
a. Supporting the perineum during delivery of the fetal head.	177 (61.0)
b. Use of episiotomy.	87 (30.0)
c. Use of assisted delivery (vacuum extraction or forceps).	1 (0.3)
d. None of the above reduce the incidence of anal sphincter injuries.	24 (8.3)
Which of the following is essential in immediate assessment of the degree of injury?	
a. A speculum examination.	56 (19.3)
b. A digital anorectal examination.	221 (76.2)
c. An endoanal ultrasound.	11 (3.8)
d. A ward hemoglobin or full blood count.	1 (0.3)
At which healthcare level should OASIS be repaired?	
a. Community health centers and district hospitals.	7 (2.4)
b. District and regional hospitals.	45 (15.5)
c. District, regional, and tertiary hospitals.	81 (27.9)
d. Regional and tertiary hospitals.	157 (54.1)
Third-degree tears may be repaired as follows?	
a. In the labor ward by a skilled midwife.	2 (0.7)
b. In the labor ward by a skilled midwife or doctor.	41 (14.1)
c. In the surgical theater by a skilled doctor.	245 (84.5)
d. Third-degree tears are not sever and only require repair if bleeding.	1 (0.3)
The most appropriate person to repair an OASIS is?	
a. A medical officer or registrar.	29 (10.0)
b. A consultant obstetrician and gynecologist.	95 (32.8)
c. The doctor with the most experience in repairing these injuries.	139 (47.9)
d. A colorectal surgeon.	26 (9.0)

*OASIS*, obstetrical anal sphincter injuries.

Either end-to-end or overlap repair was considered most appropriate by 41.7% of HCWs, with polyglactin being the preferred suture material in 47.6% of cases and 10.7% of HCWs suggesting the use of hemostatic sutures. Moreover, 43% of HCWs considered 6 hours as the optimal time in which repair should take place, whereas 6.6% of HCWs indicated that timing of repair was of no significance (Table 4).

**Table 4:** Methods of repair and postoperative care

Methods	n (%)
What is the most important prerequisite for adequate repair of an OASIS?	
a. Adequate lighting.	6 (2.1)
b. Adequate analgesia.	8 (2.8)
c. Adequate skill.	22 (7.6)
d. All of the above.	254 (87.6)
Most suitable suture material for an OASIS repair?	
a. Nylon.	28 (9.7)
b. Polyglactin (Vicryl).	138 (47.6)
c. Polydioxanone.	68 (23.4)
d. Catgut (chromic).	50 (17.2)
What is the most appropriate technique for repair of the anal sphincter muscle?	
a. End-to-end repair.	79 (27.2)
b. Overlap repair.	54 (18.6)
c. Application of hemostatic sutures.	31 (10.7)
d. Either end-to-end or overlap repair.	121 (41.7)
What is the ideal timing of repair for postinjuries?	
a. Immediately after delivery.	119 (41.0)
b. Within 6 h after sustaining injury.	127 (43.8)
c. Within 6 to 12 h after delivery.	24 (8.3)
d. The timing of repair is of no significance.	19 (6.6)
Following the repair of the injury, patients should:	
a. Be put on antibiotics and provided with stool stiffeners.	90 (31.0)
b. Be given stool stiffeners but not antibiotics as these have not shown to be of benefit.	19 (6.6)
c. Be put on antibiotics, be provided with stool stiffeners, and be referred to the physiotherapist for pelvic floor exercises.	150 (51.7)
d. Be advised to avoid intercourse for 6 wk as this may cause breakdown of the repair.	31 (10.7)
Patients who have had an obstetrical anal sphincter repair should be followed up:	
a. 1 wk after repair.	141 (48.6)
b. 6 to 12 wk after repair.	138 (47.6)
c. 6 mo after the repair.	2 (0.7)
d. No follow-up is required, provided the repair was deemed adequate.	6 (2.1)
Patients with OASIS should be advised that in future pregnancies:	
a. They should have a cesarean delivery as the risk of recurrence is high.	55 (19.0)
b. They may be allowed to have a vaginal delivery, but an episiotomy must be performed.	82 (28.3)
c. They should ideally have specialist investigations, such as endoanal ultrasound and manometry, to assess their suitability for vaginal delivery.	115 (39.7)
d. The choice of cesarean delivery or vaginal delivery should be left to the patient.	36 (12.4)

*OASIS*, obstetrical anal sphincter injuries.

Regarding postrepair management, 51.7% of respondents reported that management should include antibiotics, stool softeners, and pelvic floor exercises, whereas 10.7% of respondents indicated that sexual activity should be deferred for at least 6 weeks. The choice of follow-up varied with 48.6% of respondents choosing 1 week for postrepair follow-up, whereas 47.6% of respondents felt that patients should be followed up 6 to 12 weeks after repair.

When responding to the mode of future deliveries following OASIS, 39.7% of HCWs suggested that specialized investigations, such as endoanal ultrasound and manometry, should be used to guide this decision, 19.0% of HCWs advocated for cesarean delivery because of the risk of recurrence, and 12.4% of HCWs reported that the choice should be left to the patient (Table 4).

Regarding training in the diagnosis and management of OASIS, 50.3% of HCWs reported that they had not received any training, 21.7% of HCWs reported confidence in diagnosing and classifying OASIS, and only 17.9% of HCWs were comfortable in managing patients with OASIS. Most HCWs (96.9%) indicated a need for more in-service training, with 46.2% of participants being uncertain whether their respective units had protocols or policies in place for the management of OASIS (Table 5).

**Table 5:** Training and confidence in managing OASIS

Training level	n (%)
Have you received training in the diagnosis and management of OASIS?	
a. Yes	137 (47.2)
b. No	146 (50.3)
Do you feel that your training regarding OASIS was adequate?	
a. Yes	57 (19.7)
b. No	173 (59.7)
How confident are you in diagnosing and classifying anal sphincter injuries?	
a. Very confident	63 (21.7)
b. Somewhat confident	115 (39.7)
c. Not confident at all	102 (35.2)
How comfortable are you managing patients OASIS?	
a. Very comfortable	52 (17.9)
b. Somewhat comfortable	99 (34.1)
c. Not comfortable at all	129 (44.5)
Do you think there is a need for more in-service training?	
a. Yes	281 (96.9)
b. No	7 (2.4)
Does your unit have a protocol or policy for the management of OASIS?	
a. Yes	51 (17.6)
b. No	101 (34.8)
c. Uncertain	134 (46.2)

*OASIS*, obstetrical anal sphincter injuries.

**Figure 1:**
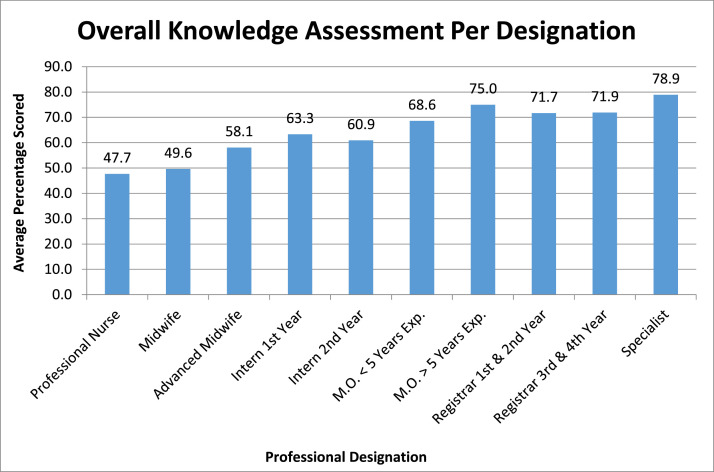
Overall knowledge assessment per designation *M.O.*, medical officer.

Using a series of 15 questions with absolute correct answers, a comparative analysis of overall knowledge revealed an average across all disciplines of 64.6%, with advanced midwives scoring an average of 58.1% and specialists an average of 78.9% (Figure 1).

Hammond. Knowledge of healthcare workers regarding obstetrical anal sphincter injuries. Am J Obstet Gynecol Glob Rep 2021.

**Figure 2:**
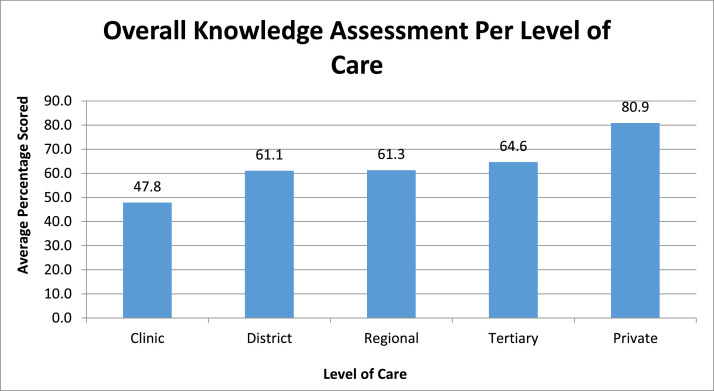
Overall knowledge assessment per level of care

A similar analysis comparing the overall knowledge at the various levels of care revealed that clinics and community health centers scored an average of 47.8%, whereas private hospitals scored 80.9% (Figure 2).

Hammond. Knowledge of healthcare workers regarding obstetrical anal sphincter injuries. Am J Obstet Gynecol Glob Rep 2021.

## Discussion

### Principal findings

Most HCWs in this study were doctors, with junior doctors forming the majority. In South Africa, most vaginal deliveries are performed by trained midwives, and junior doctors remain the first port of call when a problem is suspected or detected. Thus, the largest proportion of OASIS will initially be diagnosed by either a midwife or junior doctor.

Here, we found that a large proportion of HCWs misclassified third-degree tears, which is in keeping with the findings of Fernando et al.^5^ This was significant as the degree of injury determines the repair method.

A study by Sultan et al^1^ found that the doctors’ and midwives’ knowledge of perineal and anal sphincter anatomy was suboptimal. In the Palestinian study of Zimmo et al,^7^ only 11.4% of physicians and 9.8% of midwives had accurate knowledge of perineal anatomy. Here, we similarly found that knowledge regarding perineal anatomy was lacking.

OASIS can have devastating effects on a women's mental, physical, and social well-being.^8^ Naidu et al^9^ demonstrated a significant reduction in both minor and major OASIS with the use of perineal support. Moreover, this was the most popular preventative strategy in our cohort. The role of episiotomy in preventing OASIS has been controversial. However, there is evidence demonstrating a reduced risk of OASIS with the performance of a mediolateral episiotomy.^10^ Furthermore, van Bavel et al^11^ showed a marked risk reduction in OASIS with the use of mediolateral episiotomy at the time of operative vaginal delivery.

The National Institute of Health and Care Excellence guidelines and the Royal College of Obstetricians and Gynaecologists (RCOG) Green-top Guidelines outline the recommended management of perineal trauma.^12^^,^^13^ The RCOG guidelines state that following vaginal delivery, anal sphincter and anorectal mucosal injuries cannot be excluded without performing a rectal examination.^13^ This was recognized as appropriate by most participants.

The literature was not explicit about at which level of care OASIS should be managed. Most participants felt that these injuries should be managed at the regional or tertiary level. This may be appropriate and prudent given the availability of specialists in these centers. Inexperienced attempts at anal sphincter repair may contribute to morbidity, especially subsequent anal incontinence; hence, the RCOG guidelines state that repairs should be performed by appropriately trained and experienced clinicians.^13^ Here, almost half of the respondents felt that the most experienced doctor should perform the repair.

The RCOG guidelines state that adequate skill, lighting, and analgesia are all prerequisites for adequate repair, and this was acknowledged by most respondents.

In our study cohort, polyglactin (Vicryl) was the most popular choice of suture material. The use of polydioxanone (PDS) sutures for repair of the anorectal mucosa should be avoided as they take longer to dissolve and may cause discomfort in the anal canal; hence, use of polyglactin (Vicryl) is recommended.^14^ There is no systematic review available to evaluate the best suture material for the repair of the external anal sphincter. The only randomized controlled trial (RCT) comparing Vicryl and PDS reported no significant difference in suture-related morbidity at 6 weeks and bowel symptoms at 6 and 12 months.^15^ There was no systematic review or randomized study available to evaluate the type of suture materials used in the repair of the internal anal sphincter. Similar to external anal sphincter repair, the use of fine suture sizes, such as 3-0 PDS and 2-0 Vicryl, may cause less irritation and discomfort.^13^

Our study showed a variation in the repair methods. A large proportion of our participants indicated that OASIS should be repaired immediately or within 6 hours after delivery. However, this may not always be possible because of delays in transfer to appropriate centers with expertise. A Cochrane review demonstrated no difference in outcomes between an end-to-end repair and an overlap repair, and therefore, the end-to-end technique can be used for all external sphincter tears.^16^

The RCOG recommends prophylactic antibiotics, use of stool softeners, and referral for pelvic physiotherapy after repair.^13^ In our study group, 51.7% of respondents identified these interventions as appropriate postoperative care. Moreover, the RCOG guidelines recommend that patients be followed up 6 to 12 weeks after repair^13^; however, a larger proportion of our cohort felt that follow-up should be at 1 week.

There was no systematic review or RCT to suggest the best method of delivery following OASIS. The risk of sustaining a further third- or fourth-degree tear after a subsequent delivery is 5% to 7%.^17^ The RCOG guidelines state that all women who have sustained OASIS in a previous pregnancy and who are symptomatic or have abnormal endoanal ultrasonography and/or manometry should be counseled regarding the option of elective cesarean delivery.^13^ Furthermore, the guidelines state that the role of prophylactic episiotomy in subsequent pregnancies is not known, and therefore, an episiotomy should only be performed if clinically indicated.^13^ Here, 39.7% of respondents felt that women with previous injuries should undergo specialist investigations, such as ultrasound and manometry, to assess suitability for vaginal delivery.

## Results

The results of our study were comparable with other international studies that highlight the limitations of HCWs regarding the diagnosis and appropriate management of OASIS.

### Clinical implications

The findings of this study have clinical implications as inappropriate management of OASIS may have devastating consequences in the short, intermediate, and long-term. Thus, the need for ongoing training was highlighted.

### Strengths and limitations

The main strength of this study was that it was performed at multiple centers at varying levels of care. The target sample size was exceeded, which improved the validity of the study. One of the main limitations of the study was that midwives working in private hospitals were not included. A larger proportion of the data were obtained from regional and tertiary hospitals. This may not be entirely appropriate as it is anticipated that most vaginal deliveries take place at clinics and district hospitals.

## Conclusions

Our study demonstrated deficiencies in knowledge and confidence of HCWs managing OASIS. From these findings, several suggestions may be made to improve patient care. Every delivery unit should have a standardized protocol for the management of OASIS. Moreover, there is a need for ongoing training, and this is particularly true for the nursing personnel and junior doctors. Further research needs to be conducted to explore the effect of training on improving HCWs’ skills and patient outcomes. Lastly, ongoing audits of clinical practice are necessary to identify gaps and develop strategies to improve clinical practice.
